# Giant hepatic hemangioma treatment with transcatheter arterial embolisation and transcatheter arterial chemoembolisation; Comparative results

**DOI:** 10.3906/sag-2102-352

**Published:** 2021-08-03

**Authors:** Özhan ÖZGÜR, Hakkı Timur SİNDEL

**Affiliations:** Department of Radiology, Faculty of Medicine, Akdeniz University, Antalya, Turkey

**Keywords:** Liver, hemangioma, angiography, bleomycin, embolization

## Abstract

**Background/aim:**

Treatment of hepatic hemangiomas is a controversial topic, and traditional treatment is surgical excision. Transcatheter arterial embolisation (TAE) and transcatheter arterial chemoembolisation (TACE) have been reported as minimally invasive treatment methods. To the best of our knowledge, there are no studies comparing use of TACE and TAE for hepatic hemangioma treatment. The aim of the study is to compare symptom resolution, size reducing effects, and complications of TACE and TAE for the treatment of giant hepatic hemangiomas.

**Materials and methods:**

A total of 104 patients underwent TACE using bleomycin, and 108 patients underwent TAE. The patients were followed-up for 2 year and follow-up images at 6 months, 12 months, and 24 months were acquired. Lesion volumes in both follow-up images were calculated. The patients were examined for any possible procedure related complications as well as the status of their initial symptoms.

**Results:**

The shrinkage period was determined to have ended after 12. month in both groups. The results of the two-way mixed ANOVA showed that there was significant main effect of procedure type (p ≤ 0.001) on hemangioma volumes. Similarly, there was a significant interaction between procedure and time (p ≤ 0.001).

**Conclusion:**

Both methods are effective in symptomatic relief in properly selected patients. However, TACE causes greater volume reduction with less pain and, therefore, is the better endovascular treatment option.

## 1. Introduction

Hemangiomas are the most common primary tumors of the liver, with a prevalence ranging from 3% up to 20% being reported in the literature [1–3]. Most lesions are asymptomatic and detected incidentally [1]. Lesions greater than 4 cm are called giant hemangiomas in which case they might be symptomatic and, thus, require treatment [1,2]. When and how to treat hepatic hemangiomas is a controversial topic, with treatment generally being considered only in cases of symptomatic lesions, hemangiomas that are progressively growing or at high risk of bleeding [3,4]. Abdominal pain or discomfort and compression findings are the most frequent indication for treatment [1–4]. Corticosteroids, radiotherapy, cytotoxic agents, and surgery have been used for treatment, with surgical resection being the traditional treatment method [1,2,5,6–9]. Transcatheter arterial embolisation (TAE) is a blocking vascularity method by a substance without biological active substance. This procedure is the basic blocking vascularity method via endovascular path. It has been suggested as a less invasive alternative treatment that can be used prior to surgery as well as by itself as the primary treatment [1,2,10]. Similarly, transcatheter arterial chemoembolisation (TACE) is another blocking method. But, for this method, a biologically active agent is used in addition to the embolization material. Bleomycin, pingyangmycin, or ethanol has been reported in the literature to treat the hemangioma of liver [1,10–13]. Even though there are case series and studies demonstrating potential utility of either method, to the best of our knowledge, there are no studies in the literature comparing the efficiency, rate of success and complications of these minimally invasive treatment modalities. Thus, the purpose of this retrospective study was to evaluate and compare symptom improvement and size reducing effects and complications of TACE and TAE for the treatment of hepatic hemangiomas.

## 2. Materials and methods

This was a retrospective study, approved by the Institutional Review Board. Between 2002 and 2018, 104 patients (56 male and 48 female patients; mean age 49.5±10.8 years) underwent TACE and 108 patients (78 male and 30 female patients; mean age 50.6±12.8 years) underwent TAE.

The patients were referred to our department with giant hepatic hemangiomas detected in the workup for abdominal pain. Complete blood counts, hemostasis parameters, kidney and liver function tests before the procedure were unremarkable.

### 2.1. Procedure details

Until 2008, symptomatic patients with giant hemangiomas were treated in our center with TAE. After this date, based on reports of TACE in the literature, we started to treat those patients with TACE using bleomycin.

Prior to both procedures, the patients were premedicated with 1.2 mg/kg methylprednisolone, 50 mg ranitidine, 50 mg tramadol, and 10 mg metoclopramide right before embolisation.

### 2.2. Treatment

**Figure 1:**
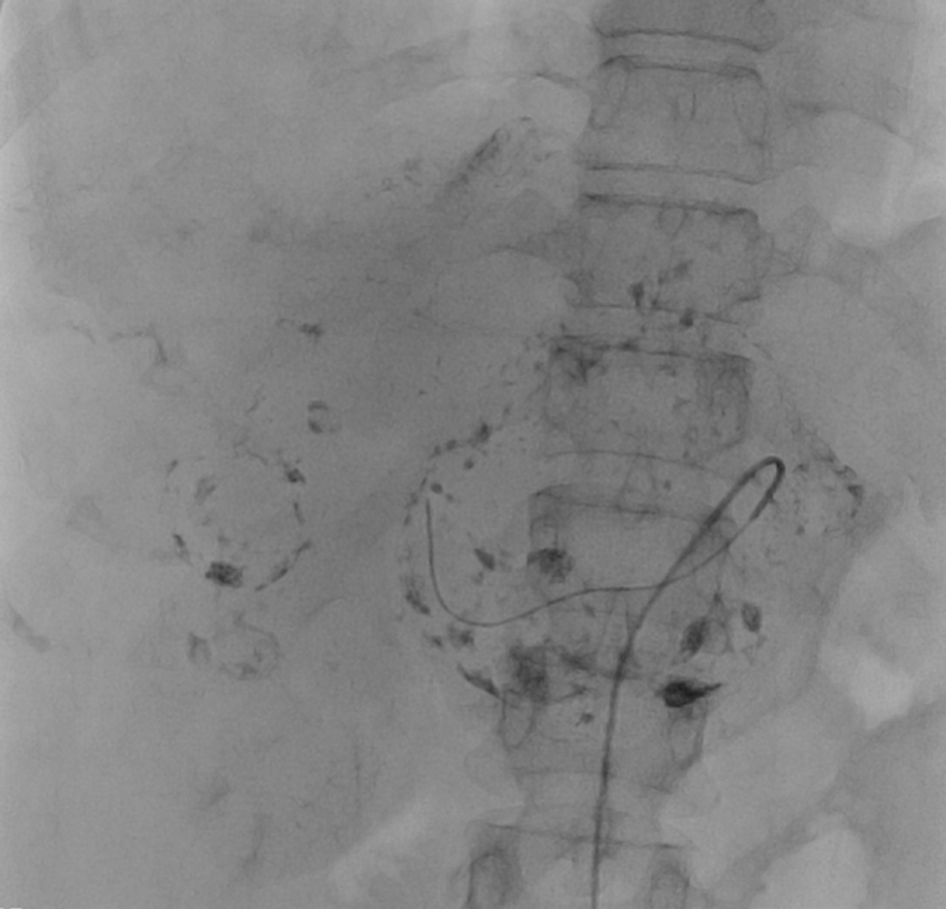
Digital subtraction angiography image showing the sinusoids of giant hemangioma filled with lipiodol-bleomycin mixture.

Embolization was performed by interventional radiologists with at least 5 years of experience. Informed consent was obtained from the patients in all cases. Under local anesthesia, femoral artery access was obtained using an 18G Seldinger needle. Abdominal aorta was catheterized with a 5F pigtail catheter (Imager, Boston Scientific, USA, COOK Medical, USA and Cordis, USA) under the guidance of a 0.035 inch J guide, and radiographs were taken. Abdominal aorta, celiac artery (CA) and superior mesenteric artery (SMA) outlet levels were detected. The hepatic arterial system was mapped, the possible variations were examined, and the nutrient artery of the existing lesion in the liver was determined. Afterwards, radiographs were taken with selectively catheterization of CA or SMA (in case of replaced hepatic artery) with 5F renal catheter (Imager, Boston Scientific, USA, COOK Medical, USA and Cordis, USA). The feeder of the hemangioma was superselectively catheterized with a 0.014inch microguide-guided (Nitrex Guidewire, ev3, Micro Therapeutics, USA) microcatheter (Prograte, Terumo, USA, HI-FLO System Kit, Boston Scientific, USA, Rebar 027 Micro Catheter, ev3, Micro Therapeutics, USA), and embolization was performed. For TAE, polyvinyl alcohol (PVA) particles (355–500 contour, Boston Scientific, USA) were used as embolizing agent. For TACE, combination of Lipiodol (Guerbet, France) with bleomycin sulphate (Bleosin-s ONKO-Koçsel, Turkey) was used as embolizing agent at 2:1 ratio in 5 mL saline solution. The procedure was terminated when as many sinusoids of the hemangioma as possible were filled, as shown in Figure 1.

In the treatment of TAE, 2 vial PVA was used in 86 of 128 hemangiomas in 108 cases, 3 vials and 3 vials in 14 patients. Second session embolization was required for 20 patients.

In TACE treatment, a 15 mL mixture was prepared by diluting 10 mL of lipiodol and 15 mg of bleomycin vials with 5 mL of isotonic (at ratio 2:1). In cases where it was needed more, this mixture was added in following proportions: 15 mL (15 mg belomycin) in 70 of 144 hemangiomas, 20 mL (20 mg bleomycin) in 30, 25 mL (25 mg bleomycin) in 25 patients and 30 mL (30 mg bleomycin) in 19 patients. The dose of 30 mg bleomycin per session was not exceeded. Second session embolization was required in 14 cases.

All second session applications were performed in cases where it was seen that sufficient reduction and clinical benefit could not be achieved at the 12th month.

### 2.3. Postprocedure

After embolisation, all patients were hospitalised for 24 h. Their body temperature, blood pressure, and heart rate were monitored at 4 h intervals. Their CBC was evaluated twice at 12 h intervals, and LFTs were evaluated after 24 h. By the results of post embolizastion syndrome, postprocedure abdominal pain was evaluated using the numeric pain rating scale, with values going from 0 to 10 as the pain increases (McCaffery Pain scale). In cases who had pain that required analgesics, in addition to the routine follow-up measures described above, their CBCs were checked, and they also underwent abdominal ultrasonography to exclude hemorrhage.

### 2.4. Calculated tumor volume

Tumor volume was calculated by using the formula 0.5 × L × W × H ( L=the greatest length, W= the greatest width, H=the greatest depth or height) at dynamic and multiphase contrast-enhanced computed tomography (CT) and magnetic resonance imaging (MRI) images, which were obtained before operation and at follow-up period.

### 2.5. Follow-up

**Figure 2:**
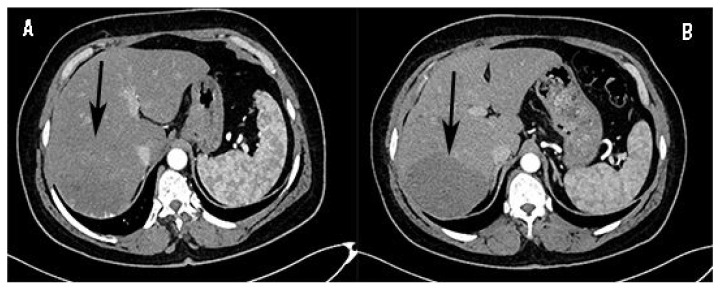
**A:** Arterial phase CT image of a hepatic giant hemangioma before TAE treatment arrow shows the lesion. **B:** Arterial phase CT image of a hepatic giant hemangioma 12 months after TAE treatment arrow shows the lesion.

**Figure 3:**
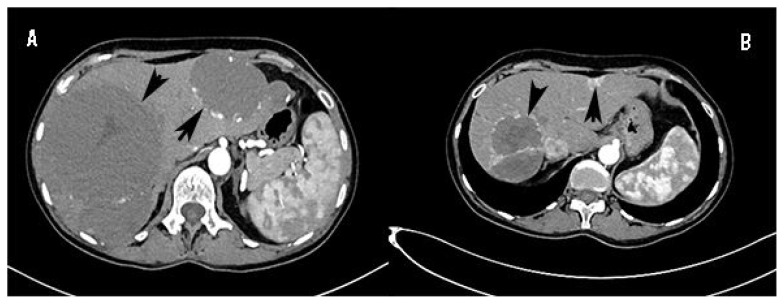
**A:** Arterial phase CT image of a hepatic giant hemangioma before TACE treantment. Arrows show the lesions. **B:** Arterial phase CT image of a hepatic giant hemangioma 12 months after TACE treatment. Arrows show the lesions.

The patients were followed-up for 2 year and follow-up images at 6 months, 12 months and 24 months were acquired as shown in Figure 2A-B and 3A-B. The patients were examined for any possible procedure related complications as well as the status of their initial symptoms. At the first year of follow-up, a second session of treatment was performed in which the lesion’s longest diameter did not fall below 4 cm or whose symptoms persisted. Other patients were removed from follow-up.

### 2.6. Statistical analysis

Statistics Package for Social Sciences (SPSS) v21.0 was used for statistical analysis. Student’s t test was used to compare means. Data waere presented as mean±standard deviation for variables suitable for normal distribution and presented as median (minimum-maximum) for variables not suitable for normal distribution. Two-factor ANOVA was used to evaluate the interaction between procedure type and time, and one-factor repeated-measures analysis of variance (ANOVA) with the use of Bonferroni adjustment for comparisons against the hemangioma volumes prior to treatment. A *p* value < 0.05 was used to assess the significance for all statistical analyses. The results are presented as means ± SEMs.

## 3. Results

In the TACE group (n = 104), 14 patients (13.5%) had 2, 6 patients (5.8%) had 3, 2 patient (1.9%) had 4 and 2 patient (1.9%) had 5 lesions; rest of the patients had one lesion for a total of 144 hemangiomas. In the TAE group (n = 108), 16 patients (14.8%) had 2 lesions and 2 patient (1.9%) had 3 lesions; rest of the patients had one lesion for a total of 128 hemangiomas.

Mean lesion volume prior to treatment (considering total volume of all lesions in patients with multiple hemangiomas) was 294.48±22.81 cm^3^ (minimum 55.00, maximum 3280.00) for TAE patients (Figure 2A) and 260.23±30.52 cm^3^ (minimum 37.00, maximum 3400.00) for TACE patients (Figure 3A). The values were similar for both groups (p = 0.526).

Following either TACE or TAE, follow-up images at 6 months demonstrated that the mean lesion volume was 183.48 ± 21.37 cm^3^ (minimum 25.00, maximum 1910.00) in TACE group and 273.50 ± 22.36 cm^3^ (minimum 45.00, maximum 2204.00) in TAE group.

In follow-up images at 12 months, mean lesion volume was 211.24 ± 17.23 cm^3^ (minimum 40.00, maximum 775.00) in TAE (Figure 2B) group and 53.38 ± 6.16 cm^3^ (minimum 5.00, maximum 590.00) in TACE group (Figure 3B).

In the 24th month follow-up, mean lesion volume was 52.13±5.94 cm^3^ (minimum 5.00, maximum 580.00) in TACE group and 208.48±17.06 cm^3^ (minimum 40.00, maximum 770.00) in TAE group.

The shrinkage period was determined to have ended after 12 month in both groups (Table). The results of the two-way mixed ANOVA showed that there was significant main effect of procedure type (p ≤ 0.001) on hemangioma volumes. Similarly, there was a significant interaction between procedure and time (p ≤ 0.001).

Findings related to lesion volumes and follow-up results are presented in Table.

### 3.1. Complications

**Figure 4:**
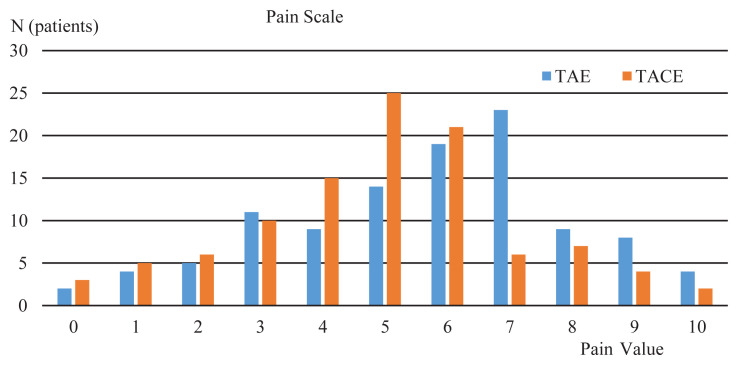
Comparison of postembolization pain symptom of two procedures according to the number of patients according to McCaffery pain scale (TAE n = 108, TACE n = 104 patients).

Following TAE, all patients had postembolization syndrome consisting of abdominal pain, nausea, loss of appetite, and low-grade fever. The most common symptom was pain. According to Mccaffery pain scale, the average score was 7.0 ± 0.8 (95% CI 6.7–7.2, minimum 4, maximum 9, median 7) (Figure 4). The patients were managed with mild analgesics. In 86 patients, the pain regressed in 24 h; however, 22 patients (20.4%) had persistent pain because of which their hospitalization was extended to 48 h. Following 48 h, they, too, were discharged upon resolution of their pain. 38 patients (35.2%) had low grade fever (<38.0˚C) not requiring medication. Three patients (5.6%) had temporary leucocytosis in the first week. Liver function tests were elevated in 48 patients (44.4%) and returned to less than twice the normal values or to normal limits in all but 12. The 12 patients where LFTs remained elevated after 24 h were followed-up for another 24 h, and their LFTs were within normal limits at the end of 48 h.

The most common complication seen after TACE was also postembolization syndrome consisting of abdominal pain, nausea, loss of appetite, and low grade fever of various severity. Average numeric pain score was 5.0 ± 1.0 (95% CI 4.7–5.2, minimum 3, maximum 7, median 5) (Figure 4). Fifteen patients (13.5%) had their postprocedural hospitalization extended to 48 h because of pain. Twenty-four patients (23.1%) had low grade fever that did not require any medication. Twenty-eight patients (26.9%) had elevated LFTs that decreased back to normal in 24 h. One patient presented with a biloma in their 1-week control and was treated with percutaneous drainage and antibiotics without any further complications. Two patients had borderline kidney functions before the procedure, and despite preventive measures before and after TACE (consisting of N-acetyl cysteine and hydration) had persisting creatinine increase at 2 months.

Postembolization pain, as graded according to the numeric pain scale, was less in the TACE group than the patients who underwent TAE (p < 0.001). The incidence of fever and LFT elevation were similar in the two groups (p = 0.358 and p = 0.060, respectively). Mean duration of postprocedural hospitalization was shorter in the TACE group (p = 0.27) and 48 h of hospitalization was more common in TAE group (p = 0.27).

There was no life-threatening complications or long-term problems (except for the patient with decreased renal function) caused by the procedures.

### 3.2. Resolution of symptoms

The procedures provided symptomatic relief in an overwhelming majority of patients. The symptoms were not resolved in 8 TACE patients, with two patients describing a similar amount of abdominal pain, one patient complaining of a decreased yet still persistent pain and one patient presenting with an increased amount of pain. It is interesting to note that in all 8 patients, lesion volume had decreased almost 4-fold. Therefore, in these cases, the initial abdominal pain was retrospectively considered not to have been caused by hepatic hemangiomas despite the lack of any other potential causes found during the workup for abdominal pain.

## 4. Discussion

First reports of TAE to treat hepatic hemangiomas come from Stanley et al. in 1983 who reported 2 infants with infantile hepatic hemangiomas treated with TAE using PVA, with shrinkage of lesions in both cases and from Allison et al. in 1985, who reported one patient with hemangioma who underwent TAE to relieve local pain and decrease risk of bleeding from rupture [14, 15]. TAE of a ruptured giant hemangioma prior to surgery was first reported in 1991 by Yamamoto et al [3,16]. Since then, in adults, TAE has been used to treat diffuse hemangiomatosis and symptomatic hemangiomas [4]. Several cases have been reported where TAE of ruptured giant hemangiomas prior to emergency surgery have been performed [17,18]. TAE has also been used to treat Kasabach–Merritt syndrome, following which coagulopathy is reported to be quickly reversed [19,20]. There are also cases where such embolization has been performed in nonruptured hemangiomas before elective surgery [21,22].

Some recent studies demonstrate TAE can be effective in treatment of symptomatic hemangiomas or lesions at risk of rupture [4]. Srivastava et al reported eight patients treated with TAE, where seven patients had complete resolution of symptoms at 9 months, and one patient had partial relief and then underwent surgery [4]. Firouznia reported 20 patients treated with PVA particles who had statistically significant reduction in lesion size and decrease in symptoms [23]. Althaus et al. reported a 29-year-old patient applying with right upper quadrant pain and revealed to have 2 giant hemangiomas, which were treated with TAE using PVL. The patient reported only minimal intermittent RUQ discomfort and was otherwise asymptomatic 2 years after the procedure [24]. Deutsch et al. reported 3 cases treated with TAE with resolution of symptoms and no significant complications [25]. The most common side effects are pain, pyrexia, leukocytosis, and nausea [4]. Serious complications are rare [4, 14]. There has been a reported case with necrosis of hemangioma followed by abscess formation [26]. Death caused by embolization of gallbladder, kidneys, and lungs with gelfoam particles has also been described [27].

Bleomycin is an agent commonly used to treat vascular anomalies [28]. It is a cycle-nonspecific cytotoxic agent that degrades DNA through direct mechanisms and also acts as an angiosclerotic agent, gradually inducing a nonspecific inflammatory response around the tumor as well as in the portal area [29]. Iodinated oil exhibits a synergistic effect. TACE with bleomycin-iodinated oil mixture (BIO) results in gradual, progressive destruction of endothelia and elimination of the pathologic vascular bed [29]. It is this quality that makes bleomycin potentially useful for treatment of hepatic hemangiomas.

Studies reporting use of TACE for hemangioma treatment are fewer in number. Interestingly, there have been cases reported where systemic use of bleomycin for cancer treatment caused a decrease in size of hepatic hemangiomas [28,30,31]. Bozkaya et al. reported 32 lesions in 26 patients treated with bleomycin. There was statistically significant reduction in size of lesions with symptomatic improvement. One of their cases had ischemic cholecystitis, otherwise there were no procedure related complications [2]. Chinese researchers reported several series using pingyangmycin, an agent similar to blemoycin. Sun et al. treated 27 patients using lipiodol mixed with pingyangmycin and reported reduction in size of lesions. [8]. Another study treated 98 patients using pingyangmycin-lipiodol emulsion with statistically significant reduction in lesion size and resolution of symptoms in all symptomatic patients [9]. Li et al. reported 836 patients treated with pingyangmycin-lipiodol mixture. Success rate as measured by resolution of symptoms was 100%. [7]. Following TACE with bleomycin or pingyangmycin, mild postembolization syndrome has been reported. TACE with BIO can cause acute liver failure, hepatic infarction, liver abscess, intrahepatic biloma, intrahepatic aneurysms, hepatic artery perforation or rupture, splenic infarction, cholecystitis, and sclerosing cholangitis [7,29]. However, most complications have been reported as case reports or cases in larger series and seem to be the exception rather than rule. Overwhelming majority of the patients reported in the above-mentioned studies did not have major complications.

Firouznia and friends found that the average diameter of tumors was 97.00 mm (range: 25–200 SD: 47.85) and 88.95 mm (range: 23–195 SD: 43.27) before and after embolization, respectively [23]. In our study, mean lesion volume prior to treatment was 294.48 ± 22.81 cm^3^ for TAE patients. In follow-up images at 12 months mean lesion volume was 211.24 ± 17.23 cm^3^ in TAE group. Results are similar with each other.

Bozkaya and friends found that the mean volume of the haemangiomas was 446.28 ± 88 cm^3^ (range 3.39–1559 cm^3^) before intervention and 244.43 ± 54.38 cm^3^ (range 94–967 cm^3^) after intervention. In our study, mean lesion volume prior to treatment was 260.23 ± 30.52 cm^3^ and in follow-up images at 12 months, mean lesion volume was 53.38 ± 6.16 cm^3^ [2]. Statistically significant reduction in size was similar in both studies.

In our study, one patient presented with a biloma in their 1-week control and was treated with percutaneous drainage and antibiotics without any further complications. In a study of 920 patients, 35 patients developed biloma, who underwent surgical hepatectomy following drainage. Biloma can develop iatrogenic, traumatic, or spontaneous. The development of biloma in the field of embolization develops due to malnutrition and infarction related to the biller tract [32].

There are several limitations of our study. First of all, the study is a retrospective study. Abdominal pain, which is the most important symptom of hemangiomas, could not be evaluated objectively before and after the procedure but could only be examined in terms of clinical benefit. In addition, the treatment response cannot be correlated with the dose of bleomycin used in the TACE procedure. It is thought that correlation of lesion volume and bleomycin dose may provide additional information in future prospective studies.

In conclusion, due to high rates of symptomatic relief and lack of major complications, both TACE and TAE are effective treatment modalities for hepatic hemangiomas in properly selected patients. To the best of our knowledge, this study is the first to compare the two methods’ efficiency in symptom resolution, incidence and severity of complications, and 2-year follow-up results. According to our findings, TACE using bleomycin causes a significantly greater reduction in lesion volume, also having milder and better tolerated postembolization abdominal pain and resulting in shorter hospitalization after the procedure. When minimally invasive endovascular treatment of giant hepatic hemangiomas is considered, TACE should be preferred over TAE.

## Figures and Tables

**Table:**
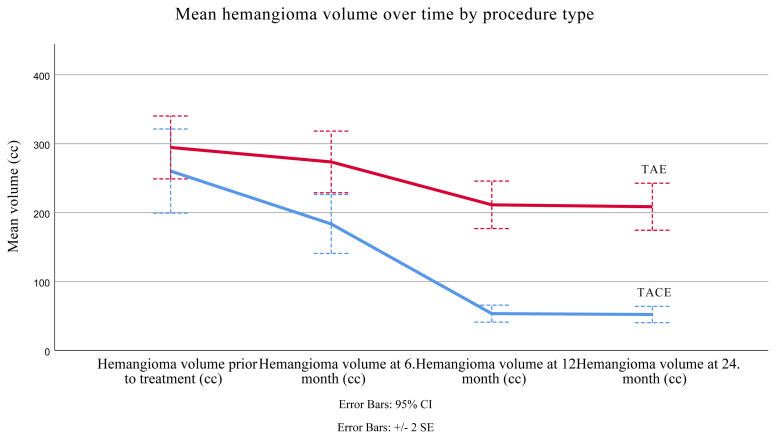
Findings related to lesion volumes and follow-up results are presented.
